# Anaesthesia for Emergency Caesarean Section in a Patient with Large Anterior Mediastinal Tumour Presenting as Intrathoracic Airway Compression and Superior Vena Cava Obstruction

**DOI:** 10.1155/2010/708481

**Published:** 2010-10-13

**Authors:** James C. S. Chiang, Michael G. Irwin, A. Hussain, Y. K. Tang, Y. T. Hiong

**Affiliations:** ^1^Department of Anaesthesiology, Queen Mary Hospital, Hong Kong; ^2^Department of Anaesthesiology, The University of Hong Kong, Hong Kong; ^3^Department of Cardiothoracic Anaesthesia, Queen Mary Hospital, Hong Kong

## Abstract

Anterior mediastinal mass is an uncommon pathology that presents significant anaesthetic challenges because of cardiopulmonary compromise. We present a case that presented in the third trimester of pregnancy with severe breathlessness, orthopnoea, and symptoms of superior vena cava obstruction. The patient had emergency Caesarean section under epidural anaesthesia with a good outcome. The paper discusses the relevant perioperative considerations for this complex scenario and reviews reports of similar conditions.

## 1. Case Report

A 34-year-old Chinese lady gravida 1 para 0, at 34 weeks' gestation, was transferred to hospital because of respiratory distress. The patient had previously been in good health until three weeks previously when she had developed progressive shortness of breath, productive cough, and headache.

Physical examination showed a 58 kg patient with an intrauterine pregnancy of 34 weeks gestation. The patient was in the respiratory distress. She was in sitting position with tachypnoea 40 breaths per minute and a peripheral oxygen saturation of 92% breathing room air. Her blood pressure and pulse rate were normal, and she was afebrile. Evaluation of the upper airway showed no signs suggestive of difficult laryngoscopy, and no hoarseness of voice. The trachea was in the midline, and the left side of the chest was dull to percussion with poor air entry. Jugular venous pressure was high, and the neck veins were engorged. The remainder of the physical examination was unremarkable.

**Figure 1:**
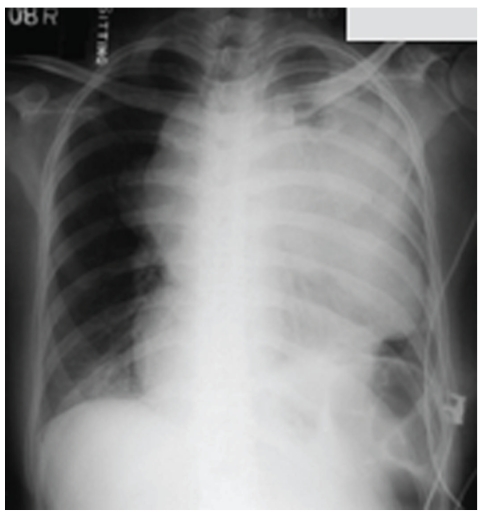
Chest X-ray was taken with abdominal shield. There was a huge mass occupying the left chest; trachea is deviated to the right. The mass caused loss of volume in left lung and elevated left hemidiaphragm.

**Figure 2:**
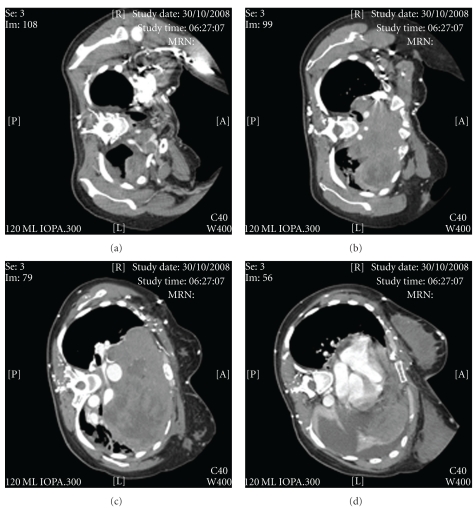
Computerised tomography of thorax in left lateral position which could be barely tolerated by the dyspnoeic patient.

An arterial blood sample breathing supplemental nasal oxygen at 2 L/min showed a pH 7.48, pO_2_ 15.0 kPa, pCO_2_ 3.8 kPa, and base excess −2 mmol/L. There was no anaemia or electrolyte abnormality. Electrocardiogram was normal. Chest X-ray (in upright position, Figure 1) and computerised tomography of thorax (in left lateral position which could be barely tolerated by the dyspnoeic patient, Figure 2) were performed on the day of admission. There was a lobulated anterior mediastinal mass sized 16.5 × 9.9 × 10 cm and extended into the left hemithorax. The mass was heterogenous in enhancement and had internal hypodense areas suggestive of necrosis. The trachea and carina were compressed against the spinal column. The left main bronchus was stenosed and accounted for volume loss and atelectasis in the lower lobe of the left lung. There was a small left pleural effusion. The heart was pushed against the diaphragm, and there was also a pericardial effusion. The superior vena cava was stretched and compressed. The left subclavian vein was dilated and tortuous, and the left brachiocephalic trunk was compressed against the sternum. Fine needle aspiration of the mediastinal mass was suspicious of malignancy, and biopsy was advised for histological evaluation. Foetal cardiotocograph showed no abnormality. At that time, the provisional diagnosis was a malignant thymoma or lymphoma.

The perioperative management was discussed in a multidisciplinary conference four hours after admission involving anaesthesiologists, obstetricians, cardiothoracic surgeons, and oncologists. Targeted treatment (e.g., radiotherapy, chemotherapy) could not be planned because there was no histological diagnosis. Caesarean section was felt to be appropriate in view of the gestational age of the foetus and the potential improvement in functional residual capacity after delivery which may reduce respiratory distress. The surgery was to be conducted under epidural anaesthesia in an operation theatre equipped with a difficult airway trolley and a cardiopulmonary bypass machine. Cardiac anaesthesiologists, cardiothoracic surgeons, and neonatologists were present during the operation.

The patient was transferred to the operation theatre six hours after admission. Acid prophylaxis with oral sodium citrate 0.3 M 30 ml and intravenous ranitidine 50 mg were given. Two 16-gauge intravenous lines were inserted in the upper and lower limbs. An 18-gauge epidural catheter was placed under aseptic technique at the L2-L3 interspace in sitting position since she could breath better. The patient was then placed in a semisitting position at 45 degrees to horizontal. Emergency femoral-femoral cardiopulmonary bypass and surgical instruments for sternotomy were prepared in case cardiorespiratory arrest occurs. The femoral arteries and veins were cannulated with 16-gauge catheters under ultrasound guidance; they could be changed to larger catheters by guidewire technique if needed. The cardiopulmonary bypass machine was primed with normal saline. Epidural anaesthesia was then established with incremental doses of ropivacaine 0.5% which were given until a sensory level of T4 was achieved bilaterally (total 150 mg over 30 minutes). Foetal heart rate was normal. The baby was delivered four minutes after skin incision, and oxytocin infusion was started at this time. Intraoperatively, she was in respiratory distress, the respiratory rate was 50 breaths per minute, but peripheral oxygen saturation maintained above 90% with 10 L/min via non-rebreathing mask, heart rate was 120–140 beats per minute, and blood pressure was normal. Blood loss was 800 ml. She received 1000 ml of a gelatin-based colloid (Gelofusine, B Braun Medical AG, Switzerland).

The patient was managed in an intensive care unit for three days postoperatively. Epidural infusion of ropivacaine 0.15% was given for analgesia. The biopsy showed it to be a germ cell tumour. She requested transfer to a private hospital for further oncology management, where she received 4 courses of bleomycin, etoposide, and cisplatin (BEP). Debulking surgery was performed four months later.

## 2. Discussion

Maternal malignancy is unusual during pregnancy (0.1%), and mediastinal tumours are particularly rare [1]. We searched MEDLINE using terms for pregnant and mediastinum tumour and anaesthesia, to look for similar cases. The majority were affected by Hodgkin's lymphoma [2–6], one by non-Hodgkin's lymphoma [7]. Hodgkin's lymphoma predominantly affects women of childbearing age, and the incidence has been reported to be 1 in 1,000 to 6,000 pregnancies [8]. The incidence is high because the peak incidence of Hodgkin's lymphoma lies in female reproductive age. Our patient had a germ cell tumour.

A mediastinal mass causes three types of intrathoracic compromise—compression of the tracheobronchial tree, compression of the pulmonary artery and heart, and superior vena cava obstruction (SVCO) [9]. The initial diagnosis of mediastinal mass is particularly difficult as signs and symptoms in the early stage are similar to common complaints during normal pregnancy [10], and there is an understandable reluctance to subject the foetus to radiation from X-rays. Dyspnoea that continues to worsen after the midtrimester is not associated with cough and that affects daily activity and orthopnoea is suggestive of this pathology [11].

### 2.1. Evaluation of Pregnant Patient with a Large Mediastinal Mass

Besides a thorough history and physical examination, a chest X-ray should be available. Computerised tomography (CT) of the thorax can assess the size of the tumour and degree of airway compression. Flow-volume loops may be performed in cooperative patients to quantify the degree of functional airway narrowing. Flexible fibreoptic bronchoscopy under local anaesthesia allows assessment of airway compression in response to changes in posture. An echocardiogram is helpful in the diagnosis of cardiac tamponade and reduction in cardiac output. However, patients with severe dyspnoea may not tolerate these procedures well.

The perioperative plan is complex and best managed by a well-coordinated multidisciplinary team. There is the dilemma of whether to have radiotherapy or chemotherapy for some shrinkage of the tumour mass before delivery. The benefits of symptom relief are often precluded by a number of maternal and foetal problems. The upward displacement of the diaphragm by the gravid uterus reduces lung size, causing the mediastinal mass to occupy most of the intrathoracic area, and radiotherapy may increase the risk of radiation-induced pulmonary damage if performed before delivery [3]. Advice about the design and use of shielding in pregnant patients is not currently available, and there is likely to be scattered radiation to the foetus [12]. Experience with chemotherapy during pregnancy has been largely in patients with breast cancer, Hodgkin's lymphoma, and leukaemia [1]. There are concerns about foetal organogenesis, growth retardation, preterm labour, and stillbirth associated with poor nutrition, weight loss, and anaemia [12]. There should be a team agreement that, in case of an emergency, maintaining the well-being of mother would maintain uteroplacental perfusion and thus the wellbeing of the foetus, therefore resuscitation of the mother has priority over the foetus. 

### 2.2. Anaesthesia Approach

Caesarean section is preferred to vaginal delivery as it avoids an increase in maternal intra-abdominal and intrathoracic pressure during contractions. Vaginal instrumental delivery with induction and augmentation of labour under full epidural anaesthesia is an option. This technique is employed in patient who cannot tolerate high intra-abdominal pressure, for example, cerebral aneurysm, severe mitral or tricuspid valve insufficiency, or pulmonary hypertension. Because vaginal delivery could be a lengthy process which could not be tolerated by the severe dyspnoeic patient, our team chose Caesarean section. 

Patient with a large mediastinal mass is a big challenge for the anaesthesiologist and that it is a third trimester pregnancy aggregates the problems. There is a high incidence of mortality and morbidity associated with general anaesthesia in patients with anterior mediastinal mass and SVCO [13]. This combination with pregnancy adds additional risk. Pregnancy-induced weight gain, upper airway oedema, and breast enlargement contribute to the possibility of a difficult airway [14]. Reduction of functional residual capacity due to pregnancy, loss of muscle tone due to general anaesthesia (with or without muscle relaxant), and further loss of lung volume from the mediastinal tumour make preoxygenation less effective. Turning the patient prone to relieve complete tracheobronchial obstruction is technically challenging. Emergency airway equipment, including a fibreoptic laryngoscope, rigid bronchoscope, and high frequency jet ventilator, should be present in the operating room, and there should be immediate access to cardiopulmonary bypass in the event of airway or cardiovascular collapse [2]. A regional rather than general anaesthetic technique is sensible to reduce the risk of potentially lethal airway collapse.

With SVCO, the use of lower extremity intravenous lines should be considered. Goh et al. [15] proposed that patients with more than 50% obstruction of the lower airway should have their femoral vessels cannulated in readiness for cardiopulmonary bypass before induction of general anesthesia. Those with less than 50% obstruction should have the femoral area prepared and draped for cannulation should the need arise. However, in pregnant patients, there is also aortocaval compression, obstructing venous return from the lower extremities. Femoral venous assess is difficult in a semisitting position, and the catheter will kink easily. An arterial line is useful for arterial blood gas measurement and direct blood measurement in the presence of reduced venous return and blood loss.

A single shot subarachnoid block is not recommended because of rapid and unpredictable hypotension and level of block in the semisitting position. An epidural catheter [16] or small incremental dose continuous spinal technique [17] allows more gradual onset of block and makes it easier to treat maternal sympathetic block [18]. With a spinal catheter, possible postdural puncture headache will be aggravated by the sitting position and could be difficult to differentiate from headache due to SVCO. Appropriate vasopressor to treat hypotension during regional anaesthesia would be phenylephrine since foetal acidosis has not been demonstrated when it is used liberally to maintain maternal blood pressure [19]. Ephedrine should be avoided because it may precipitate palpitations, and tachyarrhythmias in the context of preexisting anxiety [20] due to shortness of breath. Performing Caesarean section in an unusual position decreases surgical exposure and is a challenge for the obstetrician. With compromised venous return, postpartum hemorrhage is poorly tolerated. Replacement of blood loss by colloid or blood should be rapid, and care should be taken to avoid fluid overload and, consequent, pulmonary oedema. Pharmacological treatment for uterine hypotonia should be administered with extreme caution, as it can cause profound cardiopulmonary disturbance. Ergot alkaloids can cause hypertension and peripheral vasoconstriction. Prostaglandin F2-alpha (carboprost) can cause arterial oxygen desaturation, pulmonary oedema, hypertension, and bronchospasm [21].
